# *p*-Coumaric Acid as An Active Ingredient in Cosmetics: A Review Focusing on its Antimelanogenic Effects

**DOI:** 10.3390/antiox8080275

**Published:** 2019-08-04

**Authors:** Yong Chool Boo

**Affiliations:** 1 Department of Molecular Medicine, School of Medicine, Kyungpook National University, Daegu 41944, Korea; ycboo@knu.ac.kr; Tel.: +82-53-420-4946; 2 BK21 Plus KNU Biomedical Convergence Program, Kyungpook National University, Daegu 41944, Korea; 3 Cell and Matrix Research Institute, Kyungpook National University, Daegu 41944, Korea

**Keywords:** *p*-coumaric acid, 4-hydroxycinnamic acid, cosmetics, skin, melanin, pigmentation, oxidative stress, inflammation, UV, phytochemical, antioxidant

## Abstract

Controlling unwanted hyperpigmentation is a major challenge in dermatology and cosmetology, and safe and efficacious antimelanogenic agents are deemed useful for this purpose. *p*-Coumaric acid is a natural metabolite contained in many edible plants, and its antioxidant activities in reducing oxidative stress and inflammatory reactions have been demonstrated in various experimental models. *p*-Coumaric acid has the optimal structure to be a competitive inhibitor of tyrosinase that catalyzes key reactions in the melanin biosynthetic pathway. Experimental evidence supports this notion as it was found to be a more potent inhibitor of tyrosinase, especially toward human enzymes, than other well-known tyrosinase inhibitors such as arbutin and kojic acid. *p*-Coumaric acid inhibited melanin synthesis in murine melanoma cells, human epidermal melanocytes, and reconstituted three-dimensional human skin models. Ex-vivo skin permeation experiments and in-vivo efficacy tests for *p*-coumaric acid confirmed its efficient transdermal delivery and functional efficacy in reducing erythema development and skin pigmentation due to ultraviolet radiation exposure. Human studies further supported its effectiveness in hypopigmentation and depigmentation. These findings suggest that *p*-coumaric acid has good potential to be used as a skin-lightening active ingredient in cosmetics. Future studies are needed to extensively examine its safety and efficacy and to develop an optimized cosmetic formulation for the best performance in skin lightening.

## 1. Introduction

Melanin synthesis is an important topic in medical and cosmetic industries owing to its relevance to photo-protection, carcinogenesis processes, and skin pigmentation disorders [1,2,3,4,5]. Skin pigmentation disorders are socially significant because they can cause mental stress and lower productivity and quality of life [6]. 

Prevention and treatment strategies for hyperpigmentation include surgical treatment (chemical peeling and laser treatment), pharmacotherapy, and cosmetic camouflage [7,8,9]. Hydroquinone is primarily used as a pharmacotherapy agent, and a combination drug including retinoid is additionally used, but it can cause adverse effects such as skin irritation, allergy, mutations, and cancer [10]. In the cosmetic industry, various active ingredients such as arbutin and kojic acid have been used to control skin pigmentation [11,12,13]. However, consumers’ satisfaction is low and more effective and safer skin lightening ingredients are still in high demand [14,15]. 

*p*-Coumaric acid (4-hydroxycinnamic acid) is a phytochemical with multiple health benefits [16,17]. Its chemical structure is very similar to that of L-tyrosine, the natural substrate of tyrosinase involved in the cellular melanogenesis in melanocytes. Recently, *p*-coumaric acid was found to be a potent and selective inhibitor of human tyrosinase [18]. Its antimelanogenic effects have been demonstrated in various experimental settings including human studies [19]. Considering the need for natural skin lightening agents in cosmetics, it is of interest to scrutinize recent literature on the biological activities of *p*-coumaric acid. This review focused on the antimelanogenic properties of *p*-coumaric acid to extensively examine its potential as an active ingredient in cosmetics. 

## 2. Skin and Pigmentation 

The skin is the outermost, largest, multi-layered organ that provides a multi-functional interface between the body and external environments [20]. Humans have varied skin color that is determined by the composition and distribution of a variety of chromophores such as melanin, hemoglobin, and carotenoids [21]. Melanin is a polymeric dark pigment synthesized in melanocytes [22]. Melanin-containing melanosomes are transferred from melanocytes to adjacent keratinocytes via dendrites, distributing melanin throughout the epidermis [23]. Melanin is not only a major determinant of the colors of skin, hair, eyes, and other tissues but also an important regulator of biological functions associated with skin homeostasis [24]. Melanin plays an important role in providing a “shield” against harmful ultraviolet (UV) radiation that can cause carcinogenesis [25]. It is known that the incidence of malignant melanoma is lower in dark-skinned than in light-skinned people [26]. Experimentally increased melanin synthesis as a result of forskolin treatment reduced the rate of UV-induced skin cancer development in mice [27]. Thus, melanin pigment is critical in protection against UV radiation and in the regulation of epidermal homeostasis associated with the behaviors of melanocytes and melanoma [3,28]. 

In addition to the genetic background of an individual, a variety of non-genetic factors such as hormonal changes, nutritional status, chronic inflammation, aging, and UV radiation affect skin pigmentation [21,29]. Dysfunctions associated with melanin synthetic mechanism cause clinically relevant pigmentation disorders, that can be congenital or acquired, skin-restricted or systemic, temporary or permanent, and hypo- or hyperpigmentation related [4,30]. Hyperpigmentation disorders include melasma, freckles, and senile lentigines, in which dark pigment is deposited abnormally, unevenly, or excessively in the skin due to either endogenous and pathophysiological factors, or exogenous and environmental factors [31]. Hyperpigmentation can also occur as a secondary phenomenon after acute inflammatory reactions induced by acne, eczema, allergies, injury, burns, drug rashes, laser procedures, or as a natural process of skin aging [32]. Hyperpigmentation is an aesthetically and clinically important disease that can cause mental stress and decrease the quality of life [6]. Thus, skin hyperpigmentation is an important issue in dermatology as well as in cosmetics.

## 3. Melanin Synthetic Pathway and Its Regulation 

The gene expression of tyrosinase, tyrosinase-related protein 1 (TYRP1), and dopachrome tautomerase (DCT) in melanocytes is directed by microphthalmia-associated transcription factor (MITF) [22,30,33]. On binding of alpha-melanocyte-stimulating hormone (α-MSH) or other proopiomelanocortin-derived peptide hormones, melanocortin 1 receptor (MC1R), a G-protein-coupled receptor, undergoes conformational change, to enhance the dissociation of the G protein subunits and activate adenylate cyclase that produces cyclic AMP (cAMP) [30,34]. Then, cAMP-responsive element binding protein (CREB) transcription factor becomes active by the action of cAMP-dependent protein kinase A (PKA) and promotes gene expression of its downstream targets including MITF [35,36,37]. The gene expression and activation process of MITF are also regulated by other mechanisms involving the **c**-kit or WNT pathways [38]. The newly expressed tyrosinase protein further undergoes post-translational modifications to the active mature form [39,40,41].

Melanin synthesis starts with the oxidation of L-tyrosine and/or L-3,4-dihydroxyphenylalanine (DOPA) to L-DOPA quinone, which are catalyzed by tyrosinase enzyme [42,43]. These enzyme reactions constitute common regulatory points of biosynthetic routes for reddish-yellow pheomelanins and brownish-black eumelanins, and thus, tyrosinase is considered to be a useful target for the control of unwanted skin pigmentation. There are multiple strategies targeting tyrosinase to control melanin synthesis: (1) modulation of tyrosinase gene expression at the transcription and translation steps, (2) modulation of the post-translational modifications of tyrosinase protein and its proteolytic degradation, and (3) modulation of the catalytic activity of tyrosinase [14,44]. A variety of natural and synthetic compounds that inhibit tyrosinase catalytic activity are reported in literature [14,45,46,47,48,49,50]. Some selected examples are as follows: (1) simple phenols such as hydroquinone, arbutin, deoxyarbutin, resorcinol, 4-n-butylresorcinol, and vanillin; (2) phenolic acids such as *p*-coumaric, caffeic acid, ferulic acid, *p*-hydroxybenzoic acid, vanillic acid, protocatechuic acid, and chlorogenic acid; (3) flavonoids such as luteolin, apigenin, baicalein, chrysin, and their glycosides; and (4) stilbenoids such as resveratrol and oxyresveratrol. 

## 4. *p*-Coumaric Acid: A Phytochemical with Multiple Biological Activities

A variety of phenolic compounds found in the plant kingdom are a group of natural antioxidants with potential benefits to human health and beauty [16,17,51]. Phenolic compounds with reducing power and free radical scavenger activity may be helpful in the prevention or alleviation of many chronic diseases caused by oxidative stress [52,53]. Coumaric acids are derivatives of cinnamic acid mono-hydroxylated at the phenyl group, and *p*-coumaric acid is the most abundant isoform. *p*-Coumaric acid is found at significant levels in many fruits, vegetables, and cereals [54,55].

It is stated that *p*-coumaric acid is a relatively potent antioxidant and a scavenger of reactive oxygen species (ROS) and free radicals [56,57]. Its antioxidant activity has been demonstrated in cultured endothelial cells exposed to high glucose and free fatty acid [58], in keratinocytes exposed to UV [59], and in lens epithelial cells exposed to hydrogen peroxide [60]. It also shows antimicrobial activity by disrupting bacterial cell membranes and intercalating the groove in bacterial genomic DNA [54,61]. Polymeric preparations containing *p*-coumaric acid showed antioxidant and antimicrobial properties, aiding in the regeneration process of wounded skin [62,63]. 

In animal models, *p*-coumaric acid decreased basal oxidative stress more effectively than vitamin E, as assessed by DNA damages in rat colonic mucosa [64]. It enhanced cardiac antioxidant capacity in rats by activating nuclear factor erythroid 2-related factor 2 (Nrf2), a transcription factor that regulates antioxidant response element (ARE)-mediated gene expression of downstream target genes, such as glutathione peroxidases [65]. 

*p*-Coumaric acid showed anti-inflammatory effects in adjuvant-induced arthritic rats, reducing the levels of tumor necrosis factor-alpha (TNF-α) and macrophage phagocytic index, while increasing serum immunoglobulin levels [66]. It further attenuated hepatotoxicity due to alcohol or acetaminophen [67,68], pulmonary inflammation due to lipopolysaccharide or cigarette smoke [51,69], and cardiotoxicity due to arsenite or doxorubicin [70,71]. 

In addition, *p*-coumaric acid has been shown to inhibit proliferation and migration of cancer cells and promote apoptotic cancer cell death, supporting its potential anticancer effects [72,73,74,75]. Its chemopreventive effects against colon cancer have been demonstrated in animal models, wherein *p*-coumaric acid reduced inflammatory reactions and increased antioxidant capacity [76,77]. 

## 5. *p*-Coumaric Acid Inhibits Catalytic Activity of Tyrosinase

Many studies have used mushroom tyrosinase as an alternative for human tyrosinase in the investigation of melanogenesis, probably because it is commercially available and shares similar enzyme activities with human tyrosinase [78,79,80]. However, there are significant differences in the amino acid sequences of human and mushroom tyrosinase [81,82]. Human and mushroom tyrosinases are inhibited similarly or differently by various inhibitors, depending on their mode of actions [83,84,85,86]. 

In 1999, *p*-coumaric acid was identified as an active constituent of ginseng leaves that inhibited mushroom tyrosinase activity in vitro [87]. In later studies, dimeric coumaroyl amides such as N,N’-di-*p*-coumaroyl-1,3-diaminopropane and N,N’-di-*p*-coumaroyl-1,3-diaminoethane inhibited mushroom tyrosinase activity as compared to dimeric feruloyl amide derivatives [88,89,90]. 

A systematic assay using mushroom, murine, and human tyrosinase preparations revealed that *p*-coumaric acid is a very selective and more potent inhibitor toward human and murine tyrosinases than toward mushroom tyrosinase [18]. *p*-Coumaric acid inhibited human and murine tyrosinases ~100 and ~10 times more strongly than kojic acid, respectively, although their inhibitory effects against mushroom tyrosinase were comparable [18]. 

In another study, using human tyrosinase expressed in human embryonic kidney 293 cells, *p*-coumaric acid was shown to be the most potent inhibitor of human tyrosinase among the various phenolic acids tested [91]. The concentrations of some phenolic acids required for 50% inhibition of the enzyme activity (IC_50_) were as follows: 3 μM *p*-coumaric acid, 120 μM *p*-methoxycinnamic acid, 200 μM cinnamic acid, 250 μM caffeic acid, and 750 μM ferulic acid. *p*-Coumaric acid was more active than *m*-coumaric acid (IC_50_, 270 μM), *o*-coumaric acid (IC_50_, 300 μM), and other tested compounds, indicating it has an optimized structure to be an effective human tyrosinase inhibitor. 

On the basis of enzyme kinetics studies, *p*-coumaric acid was classified as a mixed type or competitive inhibitor of human tyrosinase depending on the substrates used: L-tyrosine or L-DOPA [18]. Given the structural resemblance to endogenous substrate L-tyrosine, *p*-coumaric acid might bind to and block the active site of the enzyme, preventing access to its substrates. 

## 6. *p*-Coumaric Acid Inhibits Cellular Melanogenesis

In 2004, Kubo et al. reported that methyl *p*-coumarate decreased melanin formation in B16 mouse melanoma cells whereas *p*-coumaric acid did not show this activity [92]. In later studies, *p*-coumaric acid inhibited melanin synthesis in B16F10 cells whereas ferulic acid showed rather melanogenic or cytotoxic effects [93]. In addition, methyl *p*-coumarate showed more potent inhibition of melanin synthesis compared to methyl ferulate [94]. 

In 2008, Park et al. tested the constituents of *Rhodiola sachalinensis* against melanin synthesis in B16F10 cells and observed that only *p*-coumaric acid inhibited melanin synthesis, whereas catechin, chlorogenic acid, and *p*-tyrosol did not show such an effect [95]. In their experiment, *p*-coumaric acid competitively inhibited tyrosinase catalytic activity but had no effect on CREB phosphorylation or tyrosinase protein expression [95]. An et al. identified *p*-coumaric acid as an active constituent of *Sasa quelpaertensis* that attenuated cellular melanin synthesis stimulated by α-MSH [96]. The authors showed that *p*-coumaric acid was more active than structurally similar caffeic acid and cinnamic acid. They further showed that *p*-coumaric acid decreased tyrosinase protein levels. 

Although there are minor inconsistencies among study results, most evidence supports that *p*-coumaric acid can attenuate cellular melanogenesis. Indeed, the antimelanogenic effects of *p*-coumaric acid were verified in later studies using human epidermal melanocytes [18,91] and 3-dimensional human skin equivalents [97]. Table 1 shows the studies on the antimelanogenic effects of *p*-coumaric acid-containing plant extracts.

## 7. Comparison of *p*-Coumaric Acid and Other Tyrosinase Inhibitors

Various plant extracts have been screened for their effects against the activities of human and mushroom tyrosinases, with the aim to identify selective inhibitors toward human tyrosinase [97,103,104]. In a study that tested 50 plant extracts, only that of *Vaccinium bracteatum* showed the strongest inhibition of human tyrosinase, followed by the extract of *Morus bombycis* [97]. Interestingly, the *Vaccinium bracteatum* extract did not significantly inhibit mushroom tyrosinase while *Morus bombycis* extract caused potent inhibition. Thus, the former extract was assumed to contain human tyrosinase-selective inhibitor which was finally identified to be *p*-coumaric acid (IC_50_, 2 μM) [97]. In-vivo efficacy of *p*-coumaric acid was evidenced by subsequent animal and human studies [19,105]. 

In another study, of the 52 medicinal plant extracts tested, strong inhibition of human tyrosinase was observed with the extracts of *Mori ramulus* and *Vitis viniferae caulis*. The former extract strongly inhibited mushroom tyrosinase activity but the latter did not. The active constituent of *Vitis viniferae caulis* responsible for the preferential inhibition of human tyrosinase was identified as resveratrol (IC_50_, 2 μM) [103]. In later studies, resveratrol and its semi-synthetic derivatives were shown to inhibit cellular melanin synthesis and showed depigmenting effects in human skin [106,107,108,109,110,111]. 

An additional screening assay of 50 marine algae extracts led to the discovery of the *Phyllospadix iwatensis* extract that inhibited human tyrosinase activity more effectively than mushroom tyrosinase [104]. Its active constituent was identified to be luteolin 7-sulfate that exhibited inhibitory effects toward human tyrosinase (IC_50_, 6 μM) but not mushroom tyrosinase. It was further found that luteolin 7-sulfate was a more potent inhibitor of human tyrosinase compared to luteolin and that the former was less cytotoxic to melanocytic cells than luteolin. Luteolin 7-sulfate also decreased the expression levels of tyrosinase in cells [112].

Compared to resveratrol and luteolin 7-sulfate, it is conceivable that *p*-coumaric acid has comparable effects on cellular melanogenesis and is relatively less cytotoxic [96,103,104]. Tyrosinase inhibition is likely not the only mechanism for antimelanogenic effects of *p*-coumaric acid, resveratrol and luteolin 7-sulfate, because they also show inhibitory effects on the tyrosinase protein expression in cells [96,106,112].

## 8. Mechanisms for Antimelanogenic Effects of *p*-Coumaric Acid

*p*-Coumaric acid did not significantly decrease the melanin levels of “unstimulated” B16/F10 cells [88,92], but its inhibitory effects on cellular melanogenesis were clearly observed in cells in the presence of α-MSH stimulation [96]. This may indicate that *p*-coumaric acid prevents “stimulated” new melanin synthesis rather than decreasing preexisting melanin. 

As inferred from the in-vitro studies, *p*-coumaric acid can reduce new melanin synthesis through direct inhibition of the catalytic activity of tyrosinase [18,95]. *p*-Coumaric acid more potently inhibited tyrosinase catalytic activity when L-tyrosine rather than L-DOPA was used as the substrate [18,95]. The structural similarity of *p*-coumaric acid to L-tyrosine suggests that the former may compete with the latter for the limited active sites on the tyrosinase enzyme.

The effect of *p*-coumaric acid on tyrosinase expression levels in cells is controversial. In some studies, *p*-coumaric acid attenuated the protein expression of tyrosinase stimulated by α-MSH [96], but other studies showed that CREB phosphorylation and tyrosinase expression were not affected by *p*-coumaric acid [95]. Interestingly, L-tyrosine is known to not only act as the substrate for tyrosinase enzyme but also play a hormone-like stimulatory role in tyrosinase gene expression. L-tyrosine enhances the binding capacity of the receptors for α-MSH [113], increasing tyrosinase gene expression [114,115,116]. It is tempting to speculate that the binding of L-tyrosine to the regulatory site on the MSH receptors may be prevented by structurally similar compounds such as *p*-coumaric acid. This could be an additional mechanism for the antimelanogenic effects of *p*-coumaric acid under certain. 

*p*-Coumaric acid was shown to suppress hydrogen peroxide-induced phosphorylation of mitogen-activated protein (MAP) kinases such as p-38, extracellular signal–regulated kinase (ERK), and c-Jun N-terminal kinase (JNK) in human lens epithelial cells [60]. It suppressed hepatic cell apoptosis by modulating the MAP kinase-signaling axis in an ROS-dependent manner [68]. *p*-Coumaric acid was also shown to alleviate cardiotoxicity and lung inflammation in animal models by scavenging ROS production and modulating Nrf2 and nuclear factor kappa B (NF-κB) signaling pathways [51,117]. Although direct evidence is currently lacking, *p*-coumaric acid has the potential to modulate redox signaling pathways associated with melanin synthesis and melanosome biogenesis. It was reported that a plant extract containing *p*-coumaric acid attenuated the gene expression of tyrosinase through modulation of the PKA/CREB/MITF pathway, although the results could not be attributed to the sole effect of *p*-coumaric acid [100]

## 9. Skin and Cell Membrane Permeability of *p*-Coumaric Acid

Enzyme-mediated melanin synthesis occurs in the melanosome of melanocytes, and a tyrosinase inhibitor should access and act on the target enzyme inside melanocytes cells, which are localized at the stratum basale of the epidermis. Thus, skin and cell membrane permeability are crucial factors that determine the in-vivo efficacy of tyrosinase inhibitors. 

Although *p*-coumaric acid is a small molecule (molar mass: 164) that may be advantageous for skin and cell membrane permeability, it has one carboxyl group that is deprotonated at neutral pH, making the compound negatively charged and decreasing cell membrane permeability. Upon direct treatment of mouse melanoma cells in vitro, *p*-coumaric acid showed weaker inhibition of melanin synthesis than methyl *p*-coumarate [92]. Although *p*-coumaric acid was a more potent inhibitor of human tyrosinase (IC_50_, 3 μM) than methyl *p*-coumarate (IC_50_, 30 μM), the former inhibited melanin synthesis less effectively than the latter in human epidermal melanocytes stimulated with L-tyrosine [105]. This phenomenon may be explained by lower cell membrane permeability of *p*-coumaric acid than methyl *p*-coumarate, as demonstrated in the assay using a hexadecane-filled membrane as a model of lipophilic cell membranes [105]. 

Excised porcine skin is a good model for the study of permeation of human skin, because they share similar histological and barrier properties [24]. Skin permeability of *p*-coumaric acid and methyl *p*-coumarate were compared using a vertical type simple diffusion device where excised porcine skin was placed between the donor and acceptor chambers [105]. *p*-Coumaric acid and methyl *p*-coumarate were separately applied in the form of semi-solid emulsion to the donor chamber, and aqueous medium in the acceptor chamber was used for the analysis of *p*-coumaric acid and methyl *p*-coumarate with high-performance liquid chromatography. The results showed that *p*-coumaric acid can pass through the skin into the underneath aqueous media, whereas methyl *p*-coumarate was captured in lipophilic skin layers or transferred into the aqueous media only after being converted to *p*-coumarate [105]. Although a hydrophobic property of a molecule is needed to enter the lipophilic layer of the skin, a hydrophilic property is also needed for diffusion out of the skin into the aqueous medium [118]. Because *p*-coumaric acid is an amphiphilic compound that possesses both hydrophobic and hydrophilic properties at neutral pH, its transdermal delivery can be faster than methyl *p*-coumarate which is very hydrophobic. 

## 10. In Vivo or Clinical Studies on the Hypopigmentation Efficacy of *p*-Coumaric Acid 

Although the antimelanogenic effects of *p*-coumaric acid were observed in cultured mammalian melanocytic cells, experiments using the animal model of zebrafish indicated that *p*-coumaric acid was not as effective as other shikimic acid pathway compounds such as shikimic acid [119]. 

When a cream containing 1.5% *p*-coumaric acid was applied on the skin of SKH-1 hairless mice, it attenuated UV-induced inflammatory responses as monitored by skin thickness and skin redness, compared to the animals treated with a control cream [120]. The effects of *p*-coumaric acid cream on UV-induced skin pigmentation were also examined in Hos:HRM-2 melanin-possessing hairless mice [105]. UV exposure of mice increased the a* values and decreased L* values, representing erythema and skin lightness, respectively. The UV-induced changes in a* and L* values were significantly more reduced in mice pretreated with creams containing 1.5% *p*-coumaric acid or methyl *p*-coumarate than those pretreated with control cream. 

In addition, *p*-coumaric acid cream was found to mitigate the UV-induced erythema and subsequent pigmentation in human skin [19]. These effects of *p*-coumaric acid cream were attributed to *p*-coumaric acid because control cream lacking *p*-coumaric acid did not show such effects. *p*-Coumaric acid cream showed a skin depigmenting effect when it was applied after human skin was fully tanned by UV [19]. Thus, the application of *p*-coumaric acid to skin, before or after sun exposure, would be beneficial in terms of mitigating UV-induced erythema and maintaining a lighter skin color. 

*p*-Coumaric acid showed no significant cytotoxicity at the effective concentration range inhibiting cellular melanin synthesis [96]. In addition, no toxic effects were observed in animal experiments and human studies [19,105]. Rhododendrol isolated from *Acer nikoense* showed antimelanogenic effects in cells, and this compound was used as a skin-brightening ingredient until its cosmetic use was stopped in 2013 because of side effects causing leukoderma and vitiligo vulgaris [121,122]. Subsequent studies found these mechanisms to be associated with these toxic effects inducing the death of melanocytes in a tyrosinase-dependent manner [123,124,125,126]. Considering the structural similarity between *p*-coumaric acid and rhododendrol, it would be interesting to compare the safety and efficacy of *p*-coumaric acid versus rhododendrol in future studies. 

## 11. Protective Effects of *p*-Coumaric Acid against UV

Solar UV radiation is a primary cause of extrinsic skin aging [127,128]. UV radiation stimulates the production of ROS and depletes endogenous antioxidants in the skin [129,130]. Natural products that provide UV-shielding and/or anti-oxidant effects are attractive cosmetic ingredients [131]. Plant extracts from *Gardenia jasminoides, Bambusae caulis in Taeniam*, and *Scutellaria baicalensis* showed protective activity against UV in vitro and in vivo [59,132,133]. 

Melanin plays an essential role in protection against UV radiation-induced skin damage, skin aging, and carcinogenesis [25], and thus artificial inhibition of melanin synthesis in the absence of UV protection may have negative effects on skin health. The inhibition of cellular melanin synthesis by small interfering RNA-mediated knockdown of tyrosinase decreased the viability of melanocytes exposed to UV [134]. In this regard, *p*-coumaric acid is an excellent candidate for dual function cosmetic agents that provide both antimelanogenic and UV-protection effects. 

There have been preliminary observations suggesting that *p*-coumaric acid may protect skin cells from UV-induced damage. Human epidermal melanocytes treated with *p*-coumaric acid before UV exposure showed significantly lesser cell death than the control cells exposed to UV or the cells treated with *p*-coumaric acid after UV exposure [18]. This phenomenon is considered melanin-independent, because *p*-coumaric acid attenuated the UV-induced cellular melanin synthesis regardless of whether it was added before or after UVB exposure. 

UV exposure of the skin induces gene expression and activation of matrix metalloproteinases (MMPs), a family of peptidases that degrade the extracellular matrix protein, thereby causing remodeling of intradermal tissue and formation of thick wrinkles [135]. Stratifin released from epidermal keratinocytes shows a paracrine effect on dermal fibroblasts, stimulating fibroblastic MMP1 gene expression by a p38 MAP kinase-dependent mechanism [136,137]. In an in vitro study, *p*-coumaric acid lowered the levels of stratifin released from the epidermal keratinocytes exposed to UV [138]. The conditioned media from epidermal keratinocytes containing different levels of stratifin stimulated MMP1 expression in dermal fibroblasts to varying degrees, indicating that *p*-coumaric acid indirectly reduced MMP1 expression in dermal fibroblasts by down-regulating stratifin expression in epidermal keratinocytes exposed to UV. 

Urocanic acid is biosynthesized from L-histidine by the action of L-histidine ammonia lyase (also called histidase) and it has been found to be a major acid-soluble, UV-absorbing compound in the stratum corneum [139,140]. Urocanic acid is considered as a “natural sunscreen”, having controversial effects on skin health [141,142,143]. *p*-Coumaric acid is similar to urocanic acid, in that it is synthesized from L-tyrosine, another aromatic amino acid, in a reaction catalyzed by L-tyrosine ammonia lyase in prokaryotes, plants, and animals [144,145]. *p*-Coumaric acid rescued the viability of HaCaT keratinocytes exposed to UV as effectively as urocanic acid in vitro [120]. Topical application of *p*-coumaric acid onto the dorsal skin of Hos:HRM-2 melanin-possessing hairless mice or SKH-1 hairless mice attenuated the inflammatory erythema responses caused by UV [105,120]. Pre-application of *p*-coumaric acid on human skin attenuated erythema due to UV exposure [19].

Like the skin, the eye is constantly exposed to light-induced photo-oxidative reactions. *p*-Coumaric acid has been shown to attenuate UV-induced oxidative damages in the eye in vitro and in vivo [146,147,148]. 

The UV-protective effects of *p*-coumaric acid may be due to the following: (1) direct UV absorption and dissipation of the absorbed energy in the form of heat, (2) multiple antioxidant actions decreasing the levels of reactive oxygen species or enhancing cellular antioxidant capacity, and (3) modulation of MAP kinase-mediated and other signaling pathways [18,58,59,120,149]. 

## 12. Conclusions and Future Directions

**Figure 1:**
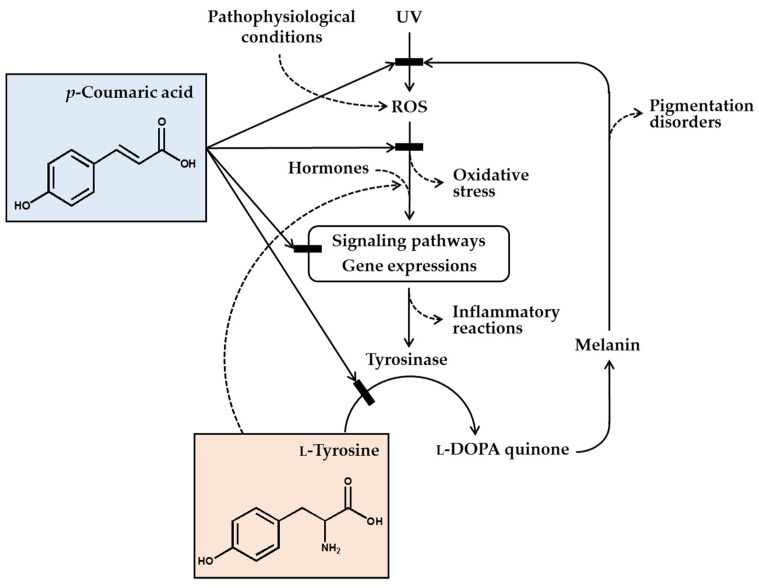
*p*-Coumaric acid can attenuate skin hyperpigmentation through multiple mechanisms. UV and other pathophysiological conditions stimulate the production of reactive oxygen species (ROS) and multiple signaling pathways leading to enhanced gene expression of tyrosinase and increased melanin synthesis. The melanin can absorb ultraviolet (UV) radiation and alleviate oxidative stress and inflammatory reactions caused by UV radiation, but the melanin deposition may cause skin pigmentation disorders. *p*-Coumaric acid has a chemical structure similar to L-tyrosine and inhibits the activity of tyrosinase, which catalyzes the oxidation of L-tyrosine and/or L-DOPA to L-DOPA quinone in the melanin biosynthetic pathway. Due to its UV absorption and antioxidant action, *p*-coumaric acid can inhibit the signaling pathways linked to gene expression of tyrosinase and inflammatory mediators. *p*-Coumaric acid can also reduce the stimulatory effects of hormones and L-tyrosine on the gene expression of tyrosinase. Thus, it is proposed that *p*-coumaric acid has advantageous biochemical properties suitable for use as a skin-lightening active ingredient in cosmetics.

In conclusion, *p*-coumaric acid has a unique chemical structure, and many of its biochemical properties are suitable for its use as a skin-lightening cosmetic ingredient. *p*-Coumaric acid inhibited the catalytic activity of tyrosinase in vitro, especially toward human tyrosinase, more effectively than other structurally similar compounds, especially when L-tyrosine was used as the substrate. *p*-Coumaric acid inhibited tyrosinase gene expression stimulated by α-MSH. Antimelanogenic effects of *p*-coumaric acid were observed in murine melanoma cells, human epidermal melanocytes, and 3-dimensional human skin equivalents. *p*-Coumaric acid also attenuated UV-induced cytotoxicity. Its skin permeability and hypopigmenting effects were shown in in ex-vivo and in-vivo experiments, respectively. The clinical outcome from human studies was also supportive for the efficacy of *p*-coumaric acid-attenuating UV-induced inflammation and subsequent pigmentation. Therefore, the antimelanogenic effects of *p*-coumaric acid in the UV-exposed skin are considered to involve multiple mechanisms: (1) absorption of UV, (2) inhibition of new synthesis of tyrosinase, and (3) inhibition of catalytic activity of preexisting tyrosinase (Figure 1). 

In addition to the melanogenesis in the melanosome, the biogenesis of melanosome and the transfer of melanosome to keratinocytes are also important steps in skin pigmentation. Currently, it is unknown whether *p*-coumaric acid has any impact on the latter two steps of skin pigmentation. Further studies are needed to address this issue and examine possible synergic effects between *p*-coumaric acid and other modulators of melanosome biogenesis and transfer. Future studies are also needed to enhance the efficacy of *p*-coumaric acid through development of the optimized formulations for efficient transdermal delivery. It is also an attractive idea to use *p*-coumaric acid in combination with other modulators of skin pigmentation, or to make hybrids between *p*-coumaric acid and other active ingredients, for the best clinical performance for skin-lightening effects [150]. Skin-lightening ingredients may be further combined with other ingredients of different functions for optimized aesthetic effects in human skin. Although *p*-coumaric acid is a natural antioxidant and has been used in cosmetics for decades, its safety should be extensively evaluated to avoid any human risk considering the long-term use of cosmetics.

## Figures and Tables

**Table 1:** Studies on the antimelanogenic effects of various plant extracts containing *p*-coumaric acid.

Literature	Plants	Additional Constituents
Park et al, 2008 [95]	*Rhodiola sachalinensis*	catechin, chlorogenic acid, *p*-tyrosol
An et al., 2008 [96]	*Sasa quelpaertensis*	-
Chao et a., 2013 [98]	*Arthrophytum scoparium*	cinnamic acid, chrysoeriol, cyanidin, catechol, caffeoylquinic acid
Jiang et al., 2017 [99]	*Panax ginseng*	protocatechuic acid, vanillic acid, salicylic acid, caffeic acid,
Choi et al., 2018 [100]	*Phyllostachys nigra*	catechin, chlorogenic acid, caffeic acid
Lee at al., 2019 [101]	*Kummerowia striata*	quercetin
Lorz at al., 2019 [102]	*Pradosia mutisii*	-
